# Adolescent and Young Adult Perceptions of Online Versus In‐Person Cognitive‐Behavioral Therapy for Eating Disorders

**DOI:** 10.1002/eat.70099

**Published:** 2026-04-12

**Authors:** Jasmine Thomas, Ava M. Pasewicz, Marley G. Billman Miller, Robert Friedberg, Jamal H. Essayli

**Affiliations:** ^1^ UT Health Austin Pediatric Psychiatry at Dell Children's Austin Texas USA; ^2^ Department of Pediatrics Penn State College of Medicine Hershey Pennsylvania USA; ^3^ Department of Psychology Auburn University Auburn Alabama USA; ^4^ Independent Training Consultant in CBT San Jose USA; ^5^ Department of Pediatrics, Department of Psychiatry & Behavioral Health Penn State College of Medicine Hershey Pennsylvania USA

**Keywords:** adolescents, anorexia nervosa, bulimia nervosa, cognitive behavioral therapy, eating disorders, online therapy, young adults

## Abstract

**Objective:**

This preliminary study investigated adolescent and young adult (AYA) perceptions of online cognitive‐behavioral therapy (CBT) for eating disorders (EDs), examining attitudes toward online versus face‐to‐face treatment and perceived effectiveness of online CBT interventions.

**Method:**

Of 258 patients contacted, 75 (29%) completed a survey and 54 (21%) were eligible for inclusion (83.3% female; mean age = 18.33 years), all of whom received telehealth services between March 2020 and December 2022. Measures assessed attitudes toward online and face‐to‐face counseling, satisfaction with online therapy, and perceived effectiveness of CBT interventions delivered online. Data were analyzed using *t*‐tests, Pearson correlations, and chi‐square tests.

**Results:**

Participants rated online CBT as significantly more valuable than face‐to‐face CBT (*t*(53) = 2.64, *p* = 0.011), with no differences in discomfort between modalities. Most participants (82.4%) reported satisfaction with online CBT, though preferences were split regarding continuing online versus in‐person treatment. Proportion of previous online sessions was negatively correlated with perceived value of online counseling (*r* = −0.43, *p* = 0.001). Participants rated most CBT interventions as equally effective online, with highest ratings for meal planning (74.0%) and cognitive restructuring (71.7%), and lowest for open weighing (35.7%).

**Discussion:**

Participants valued online CBT during the pandemic and viewed most CBT components as acceptable when delivered virtually, though individual preferences varied. Interventions requiring physical presence (e.g., open weighing) were perceived as less suitable for online delivery. While these preliminary findings suggest potential benefits of offering flexible treatment modalities, the low response rate limits generalizability and necessitates further research to examine the long‐term viability of online CBT for AYAs with EDs.

## Summary

- Adolescents and young adults with eating disorders who received treatment during the pandemic rated online cognitive‐behavioral therapy (CBT) as significantly more valuable than face‐to‐face therapy, with 82% reporting satisfaction with telehealth treatment; though low response rate (21%) limits generalizability.
- Most CBT interventions (e.g., meal planning, cognitive restructuring, psychoeducation) were perceived as acceptable whether delivered online or in‐person.
- Interventions requiring physical presence (open weighing, exposure therapy, family sessions) were perceived as less suitable for online delivery.
- Patient preferences for continuing online versus in‐person treatment were evenly split, highlighting the importance of considering individual preferences when offering treatment modalities.

## 1 Introduction

Eating disorders (EDs) disproportionately affect adolescents and young adults (AYAs), with incidence rates rising since the COVID‐19 pandemic (Gilsbach et al. 2022; Hartman‐Munick et al. 2022; Trafford et al. 2023). While treatment programs initially transitioned to remote delivery as a pandemic necessity (Murphy et al. 2020), online therapy remains widespread post‐pandemic (Ferguson et al. 2024). Evidence suggests comparable ED symptom improvements whether treatment is online or in‐person (Drury et al. 2025; Levinson et al. 2021; Ortiz et al. 2023; Raykos et al. 2021; Steiger et al. 2022). However, patient perspectives about online therapy vary. Some studies report neutral to positive perceptions (Linardon et al. 2020), while others document stronger preferences for in‐person sessions (Lewis et al. 2021; Stewart et al. 2021) or identify both advantages and disadvantages (Leder et al. 2025).

Despite guidelines for online cognitive‐behavioral therapy (CBT) for EDs (Couturier et al. 2020; Murphy et al. 2020; Waller et al. 2020), its acceptability among AYAs remains insufficiently explored, particularly regarding specific CBT interventions. Certain interventions (e.g., education) may translate more effectively to online formats than others (e.g., open weighing). This study examined AYAs' perceptions of online CBT for EDs—specifically assessing its perceived value, comfort level, and satisfaction—while also determining whether participants viewed individual CBT interventions as equally effective online versus in‐person.

Given that adolescents may prefer in‐person over online therapy (Lewis et al. 2021; Stewart et al. 2021), we hypothesized that AYAs would perceive in‐person CBT as more valuable and comfortable than online CBT. Nevertheless, since most adolescents find online therapy acceptable despite such preferences (Ahmadiankalati et al. 2020; Pretorius et al. 2009; Stewart et al. 2021), we predicted that AYAs would report satisfaction with online CBT. Given evidence that adolescents reported stronger preferences for in‐person therapy than their parents (Stewart et al. 2021), we hypothesized that favorable attitudes toward online therapy would be negatively associated with younger age. We also predicted that the proportion of online sessions would positively correlate with favorable attitudes. Finally, based on evidence supporting the acceptability of online CBT for EDs (Ahmadiankalati et al. 2020; Pretorius et al. 2009), we expected participants to rate CBT interventions as equally effective online versus in‐person.

## 2 Methods

### 2.1 Participants and Procedures

Participants were recruited from the Penn State Adolescent Medicine and Eating Disorders Clinic if they received telehealth services between March 2020 and December 2022. Of 258 patients contacted via email or in person, 75 (29%) completed a survey and 54 (21%) were eligible for inclusion. Participants completed electronic consent through REDCap; parental consent was waived given low risk. Enrollment and measure completion occurred from April 2021 to December 2022, with participants completing surveys reflecting their past treatment experiences. The clinic uses a flexible CBT model with parent collateral sessions, incorporating CBT‐consistent strategies for family support (e.g., psychoeducation, meal support) rather than formal family‐based treatment (Essayli and Vitousek 2020). Participants received a $5 gift card. This study was approved by the Penn State College of Medicine Institutional Review Board.

To be eligible, participants must have completed at least one online session. Twenty‐one participants were excluded for: receiving only in‐person services (*n* = 5), age over 30 years (*n* = 2), multiple survey completions (*n* = 2), or completing less than 50% of questions or failing the validity check (*n* = 12). The final sample (*N* = 54) was predominantly White (90.7%) and female (83.3%) (Table 1), with a mean age of 18.33 years (range: 12–28). Self‐reported diagnoses included: anorexia nervosa (68.5%), bulimia nervosa (3.7%), binge‐eating disorder (5.6%), avoidant/restrictive food intake disorder (13.0%), and other specified feeding or eating disorder (9.3%).

**TABLE 1:** Participant demographics.

Variables	Frequency (*n*)	%
Gender		
Female	45	83.3
Male	5	9.3
Other	2	3.7
Prefer to not describe	2	3.7
Race/ethnicity		
White	49	90.7
Asian/Pacific Islander	1	1.9
Hispanic/Latinx	2	3.7
Other	2	3.7
Eating disorder diagnosis		
Anorexia nervosa	37	68.5
Bulimia nervosa	2	3.7
Binge‐eating disorder	3	5.6
Avoidant/restrictive food intake disorder	7	13.0
Other specified feeding or eating disorder	5	9.3

Treatment format availability evolved with pandemic restrictions. All programs transitioned to virtual delivery in March 2020. Outpatient therapy offered format choice by June 2020. The Partial Hospitalization and Intensive Outpatient Programs (PHP/IOPs) varied: the adult PHP/IOP operated a hybrid model July–November 2020 before returning to fully virtual through Summer 2021; the child PHP/IOP remained fully virtual until July 2021. The child PHP/IOP transitioned back to in‐person by July 2021, followed by the adult PHP/IOP in September 2021. All participants retained the same therapist across transitions.

Service combinations varied: 50.9% received both PHP/IOP and outpatient CBT, 22.6% received PHP/IOP only, and 26.4% received outpatient only. Proportion of online sessions also varied: 33.3% received mostly in‐person therapy (10%–30% online), 24.1% had similar exposure to both (40%–60% online), 20.4% received mostly online therapy (70%–90% online), and 22.2% received exclusively online therapy.

### 2.2 Measures

#### 2.2.1 Demographic Questionnaire

Participants reported age, race/ethnicity, gender (male, female, other, prefer not to say), ED diagnosis, and proportion of online versus in‐person sessions.

#### 2.2.2 Online Counseling Attitudes Scales

The Online Counseling Attitudes Scales (OCAS) (Rochlen et al. 2004) evaluates attitudes toward online counseling with two subscales: Value (OC‐V) and Discomfort (OC‐D). Items use a 6‐point Likert scale (1 = *strongly disagree* to 6 = *strongly agree*). Higher scores indicate greater perceived value and discomfort, respectively. Both subscales demonstrated good internal consistency: OC‐V (*α* = 0.759) and OC‐D (*α* = 0.821).

#### 2.2.3 Face‐to‐Face Counseling Attitudes Scales

The Face‐to‐Face Counseling Attitudes Scale (FCAS) (Rochlen et al. 2004) mirrors the OCAS with “face‐to‐face” substituted for “online.” Both the Value (FC‐V) and Discomfort (FC‐D) subscales demonstrated good internal consistency: FC‐V (*α* = 0.895) and FC‐D (*α* = 0.885).

#### 2.2.4 Client Satisfaction Questionnaire (CSQ‐8)

The CSQ‐8 (Larsen et al. 1979) assessed satisfaction with online psychotherapy using eight items rated on a 4‐point scale (1 = *low satisfaction* to 4 = *high satisfaction*; total scores: 8–32). Scores were dichotomized at the midpoint: > 20 = *satisfied*, ≤ 20 = *dissatisfied*. The CSQ‐8 demonstrated excellent internal consistency (*α* = 0.962).

#### 2.2.5 Perceptions of CBT Interventions for EDs

We developed 10 items assessing perceived effectiveness of online CBT interventions: education about EDs, discussing motivation to recover, open weighing, meal planning, reviewing food records, cognitive restructuring, exposure therapy, rewards plan, family sessions, and therapeutic alliance. Participants rated whether each was as effective online as in‐person (1 = *strongly disagree* to 5 = *strongly agree*), with a “not applicable” option.

### 2.3 Data Analysis

We used IBM SPSS 30.0. Within‐samples *t*‐tests examined differences in perceived value and discomfort between online and face‐to‐face counseling. Pearson correlations assessed relationships between age, proportion of online therapy, and attitudes. Chi‐square tests explored whether satisfaction and perceived effectiveness ratings differed from chance. Sensitivity analyses excluded participants with exclusively online therapy to evaluate potential biases from differential treatment exposure.

Post hoc power analyses using G*Power 3.1.9.7 (Faul et al. 2007) with standard effect size estimates (Cohen 1992) and *α* = 0.05 showed: for *t*‐tests, power was 30.3%, 95.0%, and 99.9% to detect small, medium, and large effects; for correlations, power was 11.1%, 60.6%, and 97.7%; for chi‐square tests (degrees of freedom = 1–4) power was 7.8%–11.1%, 38.6%–59.7%, and 85.2%–95.7%.

## 3 Results

### 3.1 Attitudes Toward Online Versus Face‐To‐Face Counseling

Contrary to hypotheses, participants reported significantly higher value with online (OC‐V *M* = 24.72) than face‐to‐face (FC‐V *M* = 22.46) counseling (*t*(53) = 2.64, *p* = 0.011, *d* = 0.36). No significant differences were found in discomfort between online (OC‐D *M* = 13.04) and face‐to‐face (FC‐D *M* = 13.81) counseling (*t*(52) = 0.97, *p* = 0.336).

### 3.2 Satisfaction With Online Therapy

As hypothesized, a significant majority (82.4%) reported satisfaction with online CBT (*χ* ^2^(1) = 21.35, *p* < 0.001). Participants were divided on the CSQ‐8 item assessing preference for continuing with online over in‐person therapy, with 47.1% preferring online (*χ* ^2^(1) = 0.176, *p* = 0.674).

### 3.3 Differences in Attitudes by Age and Proportion of Online Sessions

Contrary to hypotheses, age was not associated with satisfaction or attitudes (OC‐V: *r* = 0.20, *p* = 0.186; FC‐V: *r* = 0.07, *p* = 0.669; OC‐D: *r* = −0.01, *p* = 0.928; FC‐D: *r* = 0.05, *p* = 0.754). Unexpectedly, proportion of online sessions was negatively correlated with value of online counseling (*r* = −0.43, *p* = 0.001), but not with FC‐V (*r* = 0.15, *p* = 0.274), OC‐D (*r* = 0.13, *p* = 0.362), or FC‐D (*r* = −0.24, *p* = 0.088).

### 3.4 Perception of CBT Interventions

A majority agreed or strongly agreed that most CBT interventions were equally effective online or in‐person, including: meal planning (74%; *χ* ^2^(3) = 18.64, *p* < 0.001), cognitive restructuring (71.7%; *χ* ^2^(3) = 15.04, *p* = 0.002), education (70.7%; *χ* ^2^(4) = 26.68, *p* < 0.001), discussing motivation to recover (70%; *χ* ^2^(3) = 13.20, *p* = 0.004), reviewing food records (59.6%; *χ* ^2^(4) = 19.70, *p* < 0.001), and developing a rewards plan (58.3%; *χ* ^2^(4) = 15.11, *p* = 0.004) (Table 2). Approximately half agreed or strongly agreed that exposure therapy (50%; *χ* ^2^(4) = 10.38, *p* = 0.034) and therapeutic alliance (51%; *χ* ^2^(4) = 11.84, *p* = 0.019) are equally effective online. Fewer than half agreed or strongly agreed that family sessions (48.6%; *χ* ^2^(4) = 10.57, *p* = 0.032) and open weighing (35.7%; *χ* ^2^(4) = 13.95, *p* = 0.007) are equally effective online.

**TABLE 2:** Participant ratings of online cognitive‐behavioral therapy (CBT) intervention effectiveness.

Variable	% (*n*)					
	Strongly disagree	Disagree	Neutral	Agree	Strongly agree	N/A
Education	1.9% (1)	5.6% (3)	14.8% (8)	37.0% (20)	16.7% (9)	24.1% (13)
Motivation to recover	0% (0)	11.1% (6)	16.7% (9)	42.6% (23)	22.20% (12)	7.4% (4)
Open weighing	11.1% (6)	27.8% (15)	11.1% (6)	24.1% (13)	3.7% (2)	22.2% (12)
Meal planning	0% (0)	9.3% (5)	14.8% (8)	46.3% (25)	22.2% (12)	7.4% (4)
Reviewing food records	1.9% (1)	16.7% (9)	16.7% (9)	37.0% (20)	14.8% (8)	13.0% (7)
Cognitive restructuring	0% (0)	7.4% (4)	16.7% (9)	40.7% (22)	20.4% (11)	14.8 (8)
Exposure therapy	5.6% (3)	20.4% (11)	13.0% (7)	27.8% (15)	11.1% (6)	22.2% (12)
Rewards plan	1.9% (1)	9.3% (5)	16.7% (9)	27.8% (15)	11.1% (6)	33.3% (18)
Family sessions	9.3% (5)	18.5% (10)	5.6% (3)	24.1% (13)	7.4% (4)	35.2% (19)
Therapeutic alliance	5.6% (3)	22.2% (12)	18.5% (10)	33.3% (18)	14.8% (8)	5.6% (3)

*Note:* Participants rated their agreement that various CBT interventions for eating disorders are as effective when delivered online as in‐person.

### 3.5 Sensitivity Analyses

To evaluate whether results were biased by participants receiving exclusively online therapy, we re‐ran analyses excluding the 22.2% with 100% online therapy. Participants continued to rate online therapy as more valuable than face‐to‐face (*t*(41) = 2.70, *p* = 0.010, *d* = 0.42), with no differences in discomfort (*t*(40) = −1.61, *p* = 0.115). Similarly, 82.1% remained satisfied (*χ* ^2^(1) = 16.03, *p* < 0.001), and preferences for continuing online versus in‐person therapy remained divided (41.0% preferring online; *χ* ^2^(1) = 1.26, *p* = 0.262).

## 4 Discussion

Contrary to hypotheses, AYAs who received a blend of in‐person and online therapy during the first 2 years of the pandemic rated online CBT for EDs as more valuable than face‐to‐face therapy. While the low response rate limits generalizability, these findings contribute preliminary evidence that some AYAs with EDs valued online CBT during the pandemic period. Our findings converge with recent research demonstrating that online treatments for EDs can yield comparable clinical outcomes to traditional in‐person approaches (e.g., Levinson et al. 2021; Ortiz et al. 2023), though further research is needed to determine whether perceived value translates to long‐term treatment engagement and outcomes beyond the pandemic context.

The negative correlation between proportion of online sessions and perceived value, alongside participants being divided in their preference for continuing online versus in‐person therapy, aligns with research documenting more negative attitudes among participants required to switch abruptly to virtual treatment (Stewart et al. 2021). Although our study design prevented examination of whether participants chose or were required to receive online therapy, these preliminary findings highlight the potential importance of considering individual treatment preferences when feasible.

To our knowledge, this is the first study to evaluate the perceived effectiveness of common CBT interventions for EDs in online versus face‐to‐face formats. Participants reported that most interventions were effective online, particularly “talk therapy” interventions requiring minimal adaptation (Backhaus et al. 2012; Murphy et al. 2020). However, participants expressed reservations about interventions requiring physical presence, including exposure therapy, open weighing, and family sessions. This pattern suggests that in vivo interventions originally designed for face‐to‐face implementation may present unique challenges online, as they rely on environmental manipulation, real‐time observation, or physical monitoring. Whether technological solutions such as wireless scales or enhanced video capabilities could address these challenges warrants further investigation.

Participants may have valued online therapy because they could continue receiving care safely during COVID‐19 (Berry et al. 2022), and these perceptions may not generalize to present day. However, research suggests attitudes about online care have changed favorably post‐pandemic, with patients more accepting of online services and preferring telemedicine as an option for its convenience (Berry et al. 2022; Crouch et al. 2022; Zhu et al. 2021).

Study limitations include small sample size, homogeneous demographics (predominantly young, white females), reliance on self‐reported ED diagnoses, and low response rate (21%), raising concerns about generalizability and response bias. Non‐responders may have included those less satisfied with treatment or with poorer outcomes, which could have inflated ratings of satisfaction and perceived effectiveness of online interventions. The anonymous survey prevented linking to clinical databases, precluding analyses of sexual orientation, ED diagnoses, pre‐post outcomes, and exposure to specific CBT interventions by format. The evolving availability of treatment format choice limits our ability to determine which participants were required versus chose online therapy. Additionally, early pandemic phases may have impacted ratings, and perceptions may not generalize to present day. Future research should recruit larger, more diverse samples from multiple settings, collect data on treatment adherence rates and which CBT interventions were delivered online versus in‐person, and examine whether attitudes persist post‐pandemic. Longitudinal designs would enable examination of how perceptions evolve over time and treatment phases.

In conclusion, this preliminary study found that AYAs with EDs valued online CBT during the pandemic period and perceived most CBT interventions as acceptable when delivered virtually, though preferences for continuing online versus in‐person treatment were evenly divided. Although the low response rate and potential response bias limit this study's generalizability, our preliminary findings suggest potential value in offering flexible treatment modalities and highlight the need for additional research examining patient preferences, treatment engagement, and clinical outcomes for online CBT beyond the pandemic context.

## Author Contributions

**Jasmine Thomas:** conceptualization, formal analysis, investigation, methodology, writing – original draft, writing – review and editing. **Ava M. Pasewicz:** writing – original draft, writing – review and editing. **Marley G. Billman Miller:** formal analysis, investigation, project administration, writing – review and editing. **Robert Friedberg:** conceptualization, methodology, supervision, writing – review and editing. **Jamal H. Essayli:** conceptualization, formal analysis, investigation, methodology, supervision, writing – original draft, writing – review and editing.

## Lived Experience Involvement Statement

No specific efforts were undertaken to involve persons with lived experience in the study design or execution, or in the preparation of this manuscript. However, the study centered on the perspectives of AYAs with EDs who had received treatment, with their survey responses forming the foundation of our findings.

## Funding

The project described was supported by the National Center for Advancing Translational Sciences, National Institutes of Health, through Grants KL2 TR002015, UL1 TR002014, and UL1 TR00045. The content is solely the responsibility of the authors and does not necessarily represent the official views of the NIH.

## Conflicts of Interest

The authors declare no conflicts of interest.

## Supporting information


**Data S1:** Supporting Information.

## Data Availability

The data that support the findings of this study are available on request from the corresponding author. The data are not publicly available due to privacy or ethical restrictions.
