# Supplementary material for: Adolescent and Young Adult Perceptions of Online Versus In‐Person Cognitive‐Behavioral Therapy for Eating Disorders
Source: Int J Eat Disord. 2026 Apr 12;59(8):1847–52. doi: 10.1002/eat.70099 (PMC13446235; doi:10.1002/eat.70099)
Supplement: Supplementary file 1 [file EAT-59-1847-s001.docx]

**Supplementary Material**

**The next set of questions ask for your opinion about different interventions that are often used to treat eating disorders. You may have only received some of these interventions in your therapy sessions. Please respond with the answer option “Not applicable as this**

**was not a part of my therapy” for all questions that include an intervention that you did not receive from your therapist at Penn State Health**

Education about eating disorders (e.g., Minnesota starvation study, medical complications of eating disorders, psychological effects of restriction) is as effective in an online psychotherapy session as it is in an in-person psychotherapy session.

Discussing motivation to recover from an eating disorder (e.g., pros and cons of recovery, talking about the impact of recovery on your future) is as effective in an online psychotherapy session as it is in an in-person psychotherapy session.

Open weighing (e.g., openly seeing and/or talking about your weight in therapy) is as effective in an online psychotherapy session as it is in an in-person psychotherapy session.

Meal planning (e.g., planning in therapy some of the foods that you will eat throughout the week) is as effective in an online psychotherapy session as it is in an in-person psychotherapy session.

Reviewing food records (e.g., bringing food records into therapy sessions, talking about thoughts that you wrote down on your food records in therapy sessions) is as effective in an online psychotherapy session as it is in an in-person psychotherapy session.

Cognitive restructuring (e.g., talking about your thoughts and beliefs about food, eating, and weight, reframing thoughts and beliefs in therapy sessions, challenging thoughts and beliefs in therapy sessions) is as effective in an online psychotherapy session as it is in an in-person psychotherapy session.

Exposure therapy (e.g., planning challenging foods to eat either during or between therapy sessions, setting up exposures to anxiety-provoking eating situations) is as effective in an online psychotherapy session as it is in an in-person psychotherapy session.

Developing and using a rewards plan (e.g., setting up a plan where you receive specified rewards or privileges for meeting certain therapy goals) is as effective in an online psychotherapy session as it is in an in-person psychotherapy session.

Family sessions (where a parent, significant other, or other person participates in the therapy session) are as effective in an online psychotherapy session as they are in an in-person psychotherapy session.

Therapeutic alliance (feeling understood and connected with the therapist) is as effective in an online psychotherapy session as it is in an in-person psychotherapy session.
